# A quality improvement project to improve the number of section 136 GP discharge summaries from the place of safety

**DOI:** 10.1192/bjo.2021.518

**Published:** 2021-06-18

**Authors:** Jemma Hazan, Mikail Ozer, Yathooshan Ramesh, Richard Westmoreland

**Affiliations:** 1 Camden and Islington Foundation NHS Trust, Barnet Enfield and Haringey MH NHS Trust; 2 Barnet Enfield and Haringey MH NHS Trust

## Abstract

**Aims:**

A Quality Improvement project with the aim to increase the number of patients discharged with a GP discharge summary from the Chase Farm Place of Safety over a 12 month time period by 50%.

**Background:**

An initial audit was conducted at Chase Farm Place of Safety (POS) to see if patients held under Section 136 of the Mental Health Act (S136) and then discharged home had a GP discharge letter completed and sent. The audit revealed that 0.02% of patients who were under S136 and discharged home did have a discharge letter sent to the GP.

As a result of the initial audit, key stakeholders were contacted, and involved in the intervention design and implementation. The intervention was introduced and all doctors working in the trust were emailed the new protocol

**Method:**

We implemented the following intervention:

If a patient was registered at a GP Practice then the nursing staff in the POS copied the entry of the discharging doctor from the electronic progress notes and pasted this in to the S136 discharge template on the electronic progress notes and this was emailed to the GP.

We informed Doctors to be aware that their entry would go out to the GP and should contain the following: Impression, Outcome/Plan, Specific Risk /Safeguarding concerns and specific management plans.

**Result:**

In the initial audit the notes of all patients discharged from the POS under S136 were reviewed over a 3 month period between November and January 2018. We found that 2 out of 89 patients (0.02%) had a completed GP summary which was emailed to the GP Practice.

After the intervention was introduced the notes were audited between July and September 2019. We found 33 out of 60 patients (55%) had a completed GP summary which was emailed to the GP Practice.

**Conclusion:**

There was an improvement of 54.8% in the number of discharge summaries. Further consideration needs to be given to improving this percentage and understanding what remaining barriers there are.

